# Strategies for the Improvement of Metal-Based Chemotherapeutic Treatments

**DOI:** 10.3390/biomedicines9050504

**Published:** 2021-05-04

**Authors:** Damiano Cirri, Francesco Bartoli, Alessandro Pratesi, Emma Baglini, Elisabetta Barresi, Tiziano Marzo

**Affiliations:** 1 Department of Chemistry and Industrial Chemistry (DCCI), Univerisity of Pisa, Via Giuseppe Moruzzi 13, 56124 Pisa, Italy; damiano.cirri@dcci.unipi.it; 2 Department of Translational Research and of New Surgical and Medical Technologies, Univerisity of Pisa, Via Risorgimento, 36, 56126 Pisa, Italy; francesco.bartoli@med.unipi.it; 3 Department of Pharmacy, University of Pisa, Via Bonanno Pisano 6, 56126 Pisa, Italy; emma.baglini@phd.unipi.it (E.B.); elisabetta.barresi@unipi.it (E.B.)

**Keywords:** metal-based drugs, cancer, drug development, inorganic chemistry, drug repurposing, antibacterial agents, bioinorganic chemistry

## Abstract

This article provides an overview of the various research approaches we have explored in recent years to improve metal-based agents for cancer or infection treatments. Although cisplatin, carboplatin, and oxaliplatin remain the cornerstones in tumor chemotherapy, the discovery and approval of novel inorganic anticancer drugs is a very slow process. Analogously, although a few promising inorganic drugs have found clinical application against parasitic or bacterial infections, their use remains relatively limited. Moreover, the discovery process is often affected by small therapeutic enhancements that are not attractive for the pharmaceutical industry. However, the availability of increasing mechanistic information for the modes of action of established inorganic drugs is fueling the exploration of various approaches for developing effective inorganic chemotherapy agents. Through a series of examples, some from our own research experience, we focus our attention on a number of promising strategies, including (1) drug repurposing, (2) the simple modification of the chemical structures of approved metal-based drugs, (3) testing novel drug combinations, and (4) newly synthesized complexes coupling different anticancer drugs. Accordingly, we aim to suggest and summarize a series of reliable approaches that are exploitable for the development of improved and innovative treatments.

## 1. Introduction

Metals and inorganic compounds have been used for medicinal purposes since ancient times [1,2]. However, only with the advent of modern medicine in the early 20th century, were metallodrugs first used on a scientific basis for the treatment of several diseases, including bacterial and parasitic infections [1,3,4]. More recently, with the rapid improvement of organic synthetic chemistry and the discovery of various organic molecules as effective antibiotics and anticancer agents, the use of metal complexes in medicine has undergone a rapid decrease [5]. This trend has been further extended due to concerns regarding their systemic toxicity [6].

Nonetheless, the approval of cisplatin by the FDA in 1978 spurred a renewed interest in the medicinal application of metal-based compounds. Accordingly, huge efforts were subsequently devoted to the study and development of improved platinum-based compounds [7]. This led to the approval of two analogs of cisplatin—carboplatin and oxaliplatin—that, at present, with their parent drug, are the only metal complexes used globally for the treatment of several solid malignancies. It can be estimated that almost 50% of clinical chemotherapy protocols for the treatment of cancers include these drugs [8]. In recent decades, in addition to platinum, many other transition metals have been exploited for the synthesis of innovative complexes both as therapeutic or imaging agents. Among these, compounds of ruthenium, gold, copper, zirconium, cobalt, and iridium have shown remarkable properties [1,9,10,11,12].

For therapeutic purposes, various considerations support the exploration of metal centers other than platinum [13,14]. Indeed, the commonly recognized mechanism for the anticancer action of cisplatin relies on the activation occurring through the release of the chloride ligands, with subsequent coordination at the level of the nuclear DNA, which eventually triggers apoptosis [15]. Conversely, the use of other metals may ensure different reactivity, geometry, and activation patterns that could produce lower side effects or that are effective in overcoming intrinsic or acquired resistance phenomena [16,17]. This is, for instance, the case of gold compounds, which preferentially target non-genomic biological binding partners, i.e., proteins such as thioredoxin reductase (TrxR) [18]. In addition, it is possible to exploit the different redox chemistry of different metals in order to finely control the complexes’ stability, which in turn is important to avoid off-target reactions [19].

Thus, due to their versatility, transition metals represent an extensive source for the discovery of novel and improved molecules for medicinal applications. In spite of this, it also appears clear that the approval of new inorganic drugs is a slow and difficult process [7].

Thousands of novel metal complexes have been synthesized and tested in recent decades, but the number of these entering advanced preclinical evaluation is far lower, and the overall outcome is extremely poor.

Accordingly, a relevant aspect for the development of novel chemotherapeutics to improve the currently used clinical protocols is to adopt reliable strategies that, in principle, could ensure a cheap and less time-consuming path toward advanced preclinical evaluations and clinical trials. In this view, on the basis of selected examples, we address four strategies we have pursued in recent years with the aim of improving metal-based chemotherapeutic treatments.

## 2. The Strategies

### 2.1. Drug Repurposing

So-called “drug repurposing” refers to a general approach in which an approved drug is tested for a different therapeutic purpose. This approach has advantages for the evaluation of a drug for which the key information is already available. This information includes the systemic and acute toxicity, the pharmacokinetic and bioavailability profiles, and other factors, such as the side effects and the chance of combination with other drugs [20]. As a consequence, it is possible to significantly reduce the cost and the time necessary for preclinical screening. The growing attention for this strategy is strictly dependent on the possibility to obtain precise mechanistic insights for the repurposed drug. Indeed, it is on the basis of this information that a selected drug can be repositioned for a novel indication.

#### The Case of Auranofin

**Figure 1:**
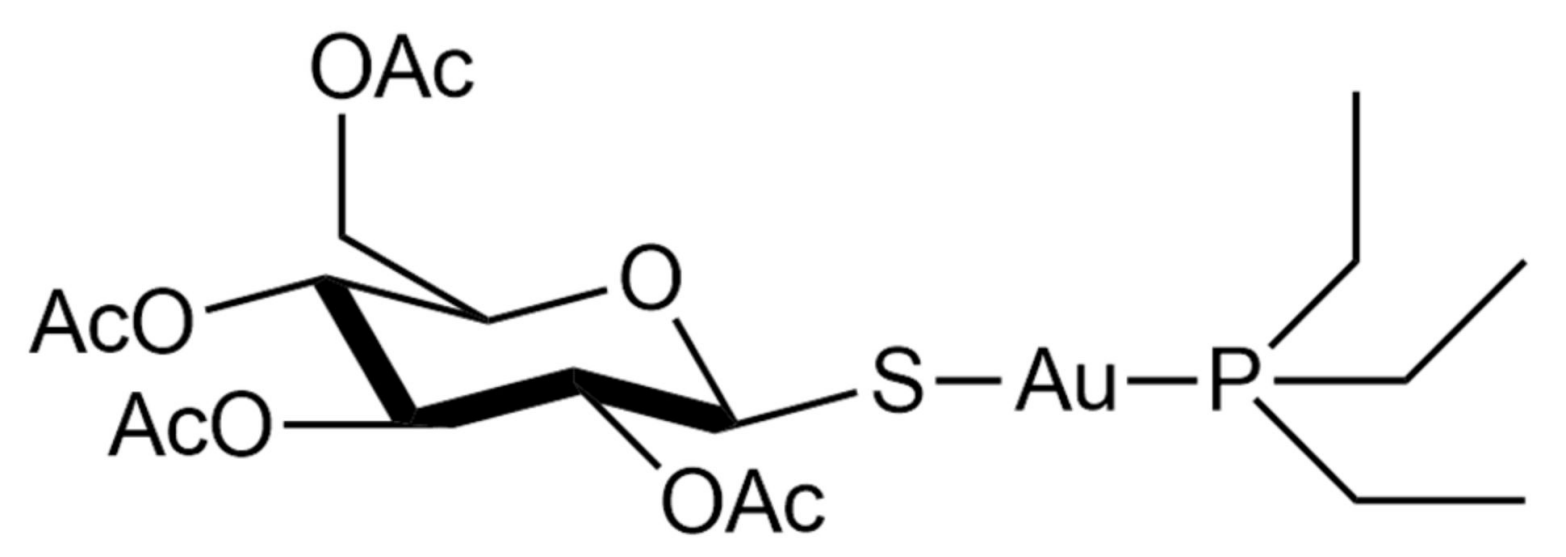
Chemical structure of auranofin [2,3,4,6-tetra-o-acetyl-L-thio-β-D-glycol-pyranoses-S-(triethyl-phosphine)-gold(I)].

Auranofin, namely, [2,3,4,6-tetra-o-acetyl-L-thio-β-D-glycol-pyranoses-S-(triethyl-phosphine)-gold(I)], is a simple gold(I) compound, orally administered as an antirheumatic agent whose brand name is Ridaura^®^ (Figure 1).

Auranofin was approved in 1985 and is reputed to be relatively safe and well-tolerated [21]. Its mode of action is not fully understood, but it possesses anti-inflammatory properties and a high affinity to the specific biological target-bearing solvent-accessible sulfur or selenium residues [22,23,24,25]. In particular, it is a strong inhibitor of the TrxR system [26]. This inhibition likely occurs through selective coordination of the gold(I) center at the level of the –Cys-Sec– redox-active motif of the enzyme [25,27,28,29]. Due to these features, it has been recently repurposed as an antiviral, antiparasitic, antibacterial, and anticancer agent [21].

In recent decades, the discovery rate of novel antibiotics has slowed and the overall number of newly approved molecules has been low. As a result, in addition to the severe problem of the emergence of bacterial strains with multidrug-resistant (MDR) and extensively drug-resistant (XDR) phenotypes, the need for improved and readily available drugs is a critical issue in order to fight this global threat [30].

The antibacterial properties of gold have been known for centuries [31], and between the 19th and 20th centuries, its dicyanide complex, K[Au(CN)_2_], was studied by the Nobel laureate Robert Koch for the treatment of tubercle bacillus infections. In subsequent years, he performed studies aiming to assess the antibacterial properties of gold and the possibility of application against several pathogens [32]. More recently, the use of auranofin has been extensively reconsidered in view of its possible repurposing as an antibacterial agent [33].

In 2013, we began to test and evaluate this drug against various strains, both Gram-positive and Gram-negative. In an early study, we evaluated auranofin against two reference strains, i.e., *E. coli* ATCC 25922 and *S. aureus* ATCC 25923, being representative of Gram-negative and Gram-positive strains, respectively [34]. The obtained results showed that auranofin possesses potent concentration-dependent antibacterial activity against *staphylococcus*, providing the opportunity for further testing against, in particular, severe bacterial infections due to resistant *staphylococci*. In the same study, we noted that auranofin was far less effective, or ineffective, in the case of the Gram-negative strain [34]. Because the reduced activity in the case of the Gram-negative model is likely the result of a reduced internalization of the complex [35,36,37], we carried out a second and more detailed study in which auranofin and a panel of its analogs bearing different ligands in place of thiosugar or silver in place of gold, respectively, were comparatively tested against clinical isolates including major Gram-positive, Gram-negative and other pathogens, such as fungal pathogens showing relevant resistance phenotypes [38]. Additionally, to gather precise information about the mechanisms responsible for the reduced activity against the Gram-negative isolates, we performed experiments using auranofin and the other complexes in the presence of a permeabilizing agent, i.e., polymyxin B nonapeptide. Based on the overall integration of the obtained results, it was noted that auranofin possesses good stability in physiological-like conditions and the presence of thiosugar is not mandatory for the pharmacological effects, confirming the previous finding that the real pharmacophore is the cationic fragment [Au(PEt_3_)]^+^. Moreover, according to findings from other groups, we were able to confirm that the lower effect in the Gram-negative strains was likely related to a decreased drug uptake [38].

An important paper by Jackson-Rosario and Self, published in 2009, reported that the antimicrobial action of auranofin against *Treponema denticola* is likely mediated by the inhibition of selenium metabolism, which is crucial for the synthesis of selenoproteins [39]. Basically, the high affinity of gold for selenium makes auranofin highly reactive toward this element. In turn, this reactivity subtracts the selenium available to the bacteria for the synthesis of key proteins with consequent inhibition of bacterial growth.

Beyond its recognized activity for the treatment of bacterial infections, auranofin entered phase II clinical trials (ClinicalTrials.gov Identifier: NCT02736968) for Giardia Protozoa (giardiasis) infection, whose treatment primarily relies on the use of 5-nitro imidazole antimicrobials such as metronidazole. However, even in this case resistance is a critical issue. Importantly, according to preclinical studies, auranofin is not only active against this pathogen, but it is also capable of overcoming resistance and is particularly active in metronidazole-resistant strains [40].

Auranofin has also been investigated for the treatment of HIV infections. In preclinical models, it was able to interfere with several phases of the viral cycle. The high susceptibly to auranofin of various subsets of cells involved in both HIV production and persistence has been demonstrated. Overall, auranofin strongly perturbs, through its pro-apoptotic effects, the activation and differentiation stages of CD4+ T lymphocytes, whose role is strictly connected to the viral production, latency, and viral reactivation [41]. Consequently, two clinical trials involving auranofin for HIV treatment have begun (ClinicalTrials.gov, Identifiers: NCT02961829 and NCT02176135). Furthermore, the antiviral activity of auranofin prompted us to suggest its evaluation for the treatment of COVID-19 infections within the wide drug repositioning program that was initiated to find effective drugs to counter the rapid spread of the pandemic [5,42].

Another field in which gold compounds, and more precisely auranofin, are being repurposed with remarkable results is that of anticancer chemotherapy [43,44,45,46,47]. Indeed, auranofin entered at least seven clinical trials as an anticancer drug for the treatment of ovarian, lung, glioblastoma, and chronic lymphocytic leukemia (ClinicalTrials.gov Identifiers: NCT01747798; NCT02126527; NCT03456700; NCT01737502; NCT02063698; NCT01419691; NCT02770378). Recently, we investigated the in vitro activity of auranofin in a panel of four colorectal cancer cell lines, namely, HCT8, HCT116, HT29, and Caco2. The compound was comparatively tested for its effects in the normal cell lines HDFa (human dermal fibroblast, adult) and HEK293 (human embryonic kidney) [28]. Results revealed that auranofin exerts a potent cytotoxic effect against these cell lines, reducing the IC_50_ values to the sub-micromolar range; in contrast, no cytotoxic effects were found in the normal cell lines, highlighting the selectivity for cancer cells. Overall, these results may warrant the assessment of auranofin in clinical trials for colorectal cancer.

Next, using high-resolution mass spectrometry, ethidium bromide displacement, and viscosity experiments, it was possible to demonstrate that the compound does not react toward single or double-stranded DNA models, strengthening the evidence that the targets for its anticancer action are likely non-genomic. This concept was further confirmed by inhibition experiments of TrxR. This enzyme is important for the maintenance of the redox balance of cells. Indeed, the impairment of the TrxR system may result in the loss of the redox homeostasis, which in turn may lead to oxidative stress and apoptosis [28]. Results from the *in vitro* screening highlighted a good correlation between the IC_50_ values for the anticancer activity of auranofin in the colorectal cancer cells and the IC_50_ for TrxR inhibition. This latter evidence can be explained based on the interaction between the [Au(PEt_3_)]^+^ cation and the TrxR redox-active site. This reaction pattern is in perfect agreement with those reported in the case of auranofin interaction towards other thiol-owning proteins and peptides [23,24].

### 2.2. Simple Modification of the Chemical Structures of Approved Metal-Based Drugs

**Figure 2:**
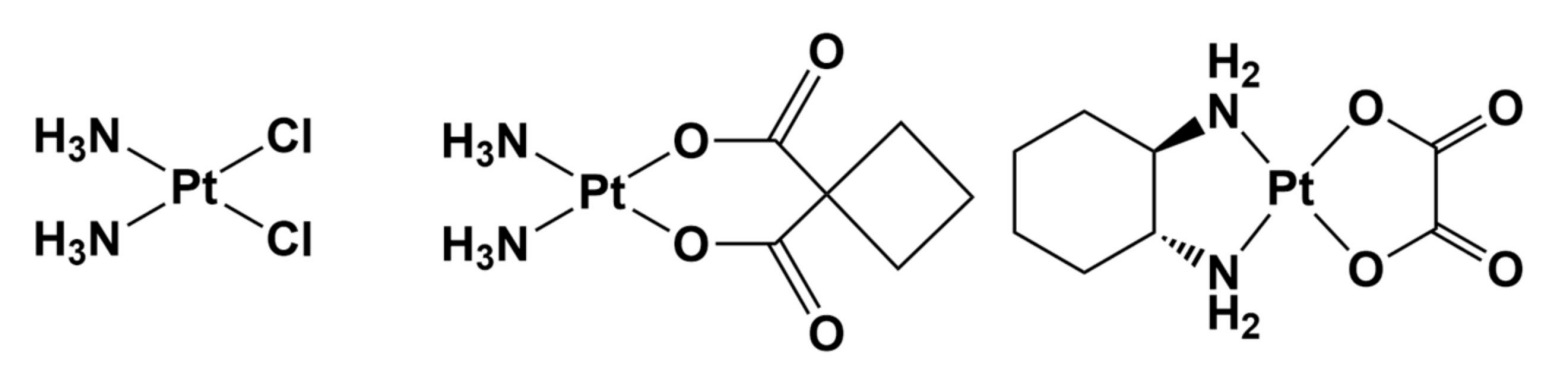
Chemical structures of (from the left) cisplatin, carboplatin, and oxaliplatin.

The examination of the chemical structures of second—and third—generation approved anticancer platinum drugs, e.g., carboplatin and oxaliplatin, quickly indicates that these important complexes have been substantially developed as cisplatin-like drugs; that is, they are cisplatin analogs (Figure 2) [7].

It thus appears that the strategy of modifying the structure of approved metal-based drugs is effective, and may be extremely helpful in the design and development of improved drugs. Replacing the ligands at the metal center not only may affect the cytotoxic potency, but also could modulate the side effects, increasing the tolerance to the new drug.

It is known that cisplatin and its analogs are prodrugs that exert their anticancer activity only after the activation step that involves the release of the labile ligand, which is chloride, cyclobutanedicarboxylate, or oxalate for cisplatin, carboplatin, or oxaliplatin, respectively [45]. Consequently, structural modifications could affect the kinetics of the release of the labile ligand and thus the activation process itself [46]. In this framework, the structural diversity of carboplatin and oxaliplatin results in a toxicity level that is lower than that of cisplatin, and particularly nephron- and ototoxicity [48,49].

Despite the similar structure of these three anticancer agents, the case of oxaliplatin is interesting. In 2017, Lippard and coworkers reported an innovative mode of action. In particular, at variance with cisplatin and carboplatin, which trigger apoptosis as a response to DNA coordination and damage, oxaliplatin does not induce cell death in response to its binding on DNA, but rather through the induction of ribosome biogenesis stress [50].

The approach of limited structural modifications of an already approved drug offers an additional advantage from a synthetic perspective (i.e., the synthesis is likely already optimized or its optimization is inherently easier), and this may increase the appeal for pharma companies in view of advanced preclinical evaluation.

For example, our research group prepared three oxaliplatin derivatives via the replacement of the oxalate anion with simple halides. These compounds showed interesting differences in their pharmacological behavior. More precisely, all of the compounds exhibited notable cytotoxicity, but their pro-apoptotic effects in some cases were found to be higher with respect to oxaliplatin itself [51,52]. Since the approval of cisplatin, thousands of analogs bearing different ligands or metal centers have been developed. Accordingly, it is impossible to summarize in this review all of the metal-based compounds that show pharmacological activity [1]; therefore, we propose here two relevant examples from our research experience that were selected as promising antineoplastic agents.

#### The Case of Cis-PtI_2_(NH_3_)_2_ and Au(PEt_3_)I

**Figure 3:**
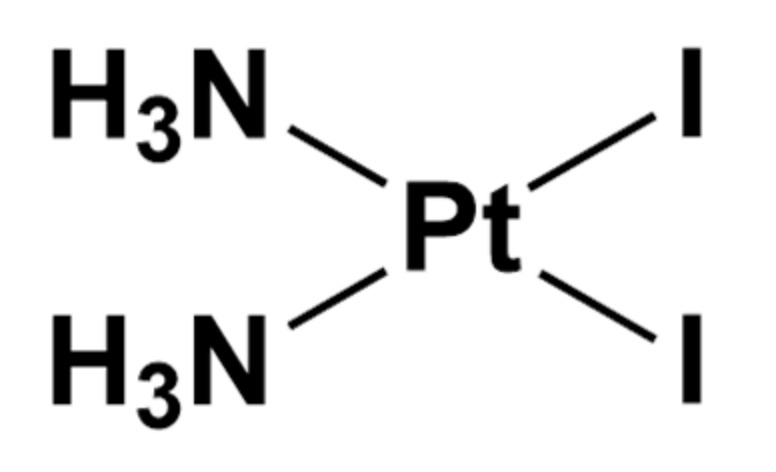
Chemical structure of cis-PtI_2_(NH_3_)_2_.

cis-PtI_2_(NH_3_)_2_, namely diamminediiodidoplatinum(II), is the iodide analog of cisplatin bearing two iodide ligands in place of chloride (Figure 3).

This complex is a key intermediate in the synthesis of cisplatin following the method of Dhara [53]. This synthetic approach exploits the strong trans influence of iodide (i.e., the ability of iodide to drive substitution trans to itself) to selectively and quantitatively afford the cis isomer [53,54]. The latter, when treated with two equivalents of AgNO_3_ and an excess of NaCl, allows cisplatin to be obtained [55].

Recently, we carried out a detailed study of this complex to assess whether pre-existing claims of inactivity as an anticancer drug—mainly based on preliminary studies carried out on in vivo sarcoma models—were reliable [54]. To our surprise, we found that this complex is not only effective in overcoming cisplatin resistance in a panel of representative cancer cell lines, but is also characterized by an unconventional reactivity toward model proteins compared with cisplatin [56].

When cis-PtI_2_(NH_3_)_2_ is reacted with hen egg-white lysozyme (HEWL)—a protein that is extensively used as a model to study the reactivity of several medicinal metal complexes because it is suitable for both mass spectrometry studies and X-ray crystallography [57,58]—in contrast to cisplatin, which reacts in a classical fashion by releasing the chloride ligands, cis-PtI_2_(NH_3_)_2_ also coordinates the proteins at the level of His15, but through the release of ammonia ligands [56]. Similar behavior was also found upon reactivity with cytochrome c. Computational details confirmed that, in contrast with cisplatin, the activation barriers for the release of the halide ligands in cis-PtI_2_(NH_3_)_2_ are lower. In turn, this could be due to the strong trans influence of iodide in comparison with chloride [59]. Conversely, cis-PtI_2_(NH_3_)_2_, reacts similarly to cisplatin when incubated with single- or double-stranded DNA models, but the metalation of DNA is less efficient in the case of cis-PtI_2_(NH_3_)_2_ than that of cisplatin [54,60]. The unconventional reactivity of cis-PtI_2_(NH_3_)_2_ toward the model proteins with respect to cisplatin prompted us to extensively evaluate the anticancer properties of this iodide analog. Unexpectedly, we found that, in contrast with the previous reports on the substantial inactivity of this molecule, its cytotoxic activity was not only comparable to that of cisplatin in vitro, but in the case of colorectal cancer line HCT-116 resistant to cisplatin, cis-PtI_2_(NH_3_)_2_ was capable of overcoming the platinum resistance. The higher cytotoxic potency in this cell line was not directly linked with an augmented cell uptake, despite the fact that cis-PtI_2_(NH_3_)_2_ is more lipophilic than cisplatin [54]. It is important to consider that these differences may be the result of a different recognition process for the two complexes. Thus, the replacement of chloride with iodide could determine a different mode of action, eventually resulting in the enhanced cytotoxic activity of the iodide analog toward resistant cancer cell lines.

**Figure 4:**
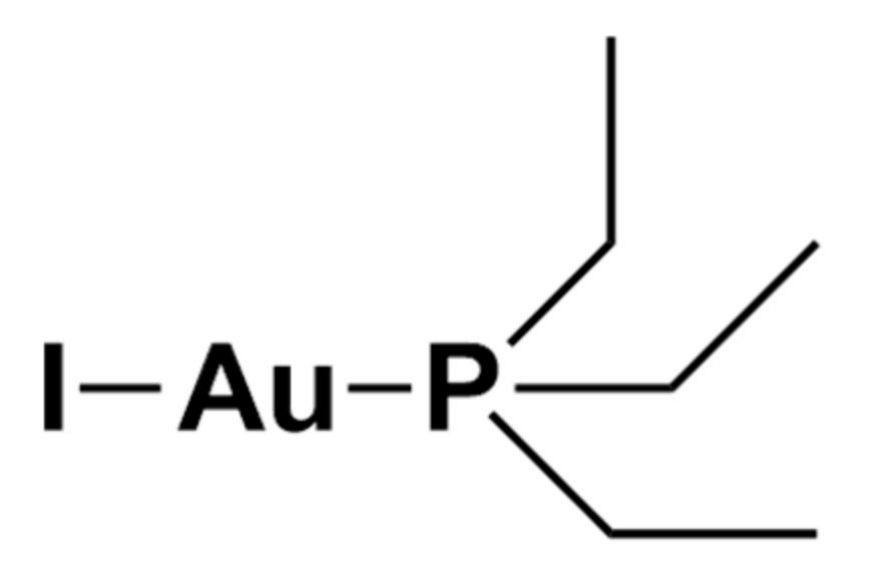
Chemical structure of Au(PEt_3_)I.

Au(PEt_3_)I is the iodide analogue of auranofin in which the thiosugar moiety is replaced by an iodide ligand (Figure 4).

In a manner similar to that of the iodide-replaced analog of cisplatin, the effect of the replacement of the thiosugar moiety of auranofin with different halide ligands was recently investigated. Subsequently, a comparative study of the manner in which this structural modification affects the overall biochemical and anticancer profile was carried out [28,61]. Among the various analogs tested and evaluated, it was found that Au(PEt_3_)I was the most promising, due to its improved anticancer properties.

The first explanation of the improved anticancer potency of Au(PEt_3_)I compared to auranofin could be linked to the higher lipophilic character of Au(PEt_3_)I. This has a LogP value of 4.6, whereas the value for auranofin is significantly lower (LogP = 1.6). This interpretation is consistent with a pilot comparative in vivo study of the gold biodistribution in mice. From this study, it was found that Au(PEt_3_)I possess generally higher accumulation than auranofin, both in organs and peripheral blood [51]. In addition, the replacement of the thiosugar ligand with the iodide, at least in principle, should not impair the pharmacological activity because the cationic pharmacophore fragment [Au(PEt_3_)]^+^ remains unaltered. Experiments on a panel of colorectal cancer cell lines (HCT8, HCT116, HT29, and Caco2) or on a representative ovarian line (A2780) indirectly confirmed that the maintained cytotoxicity of Au(PEt_3_)I is comparable to that of auranofin in vitro (sub-µM range) [28,61]. In addition, in analogy with auranofin, the potency in inhibiting the TrxR enzyme is in good agreement with the IC_50_ values in the cell lines, indirectly supporting the TrxR system as a likely target for the anticancer action of Au(PEt_3_)I.

Further interesting results for the anticancer potency of Au(PEt_3_)I were obtained in two in vivo ovarian cancer models, i.e., xenograft subcutaneous and orthotopic models. However, prior to performing experiments on the anticancer activity in mice, tolerability studies were carried out to assess the in vivo safety of Au(PEt_3_)I. The complexes were well tolerated at two different doses (20 and 40 mg/kg). In a subsequent preliminary study, the biodistribution of the drug was also investigated in comparison with auranofin, highlighting a higher accumulation of the iodide analog both in organs and blood [61].

**Figure 5:**
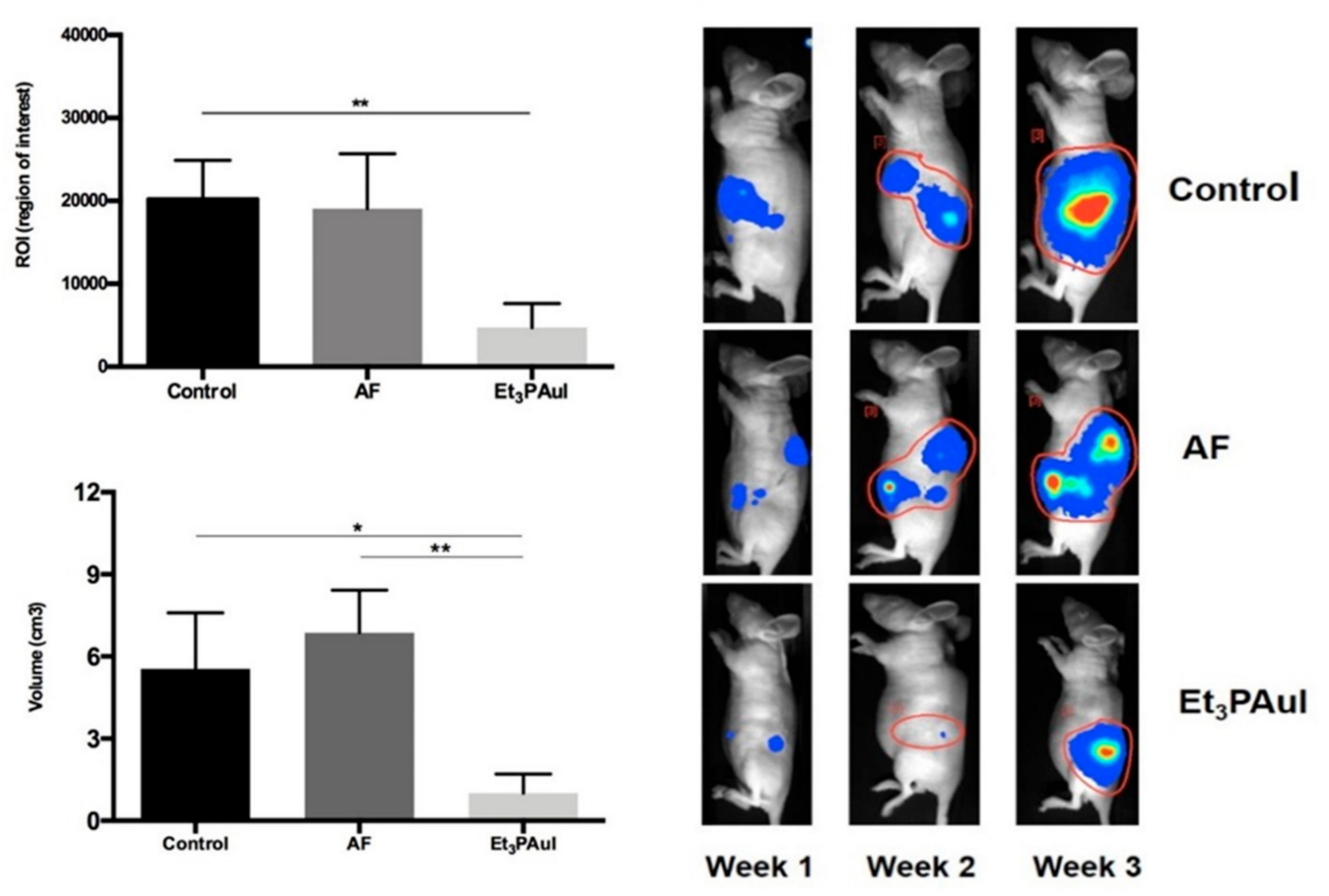
The orthotopic model was established by injection of 1 × 10^6^ A2780-luc cells in the ovary bursa of nude mice. Left Panel (upper corner): Levels of ROI after 1 week of treatment; data are reported as the mean of each group (*n* = 10 animals/group) ± SD (** *p* < 0.01); (bottom) volume of tumor masses (mean ± SD) at sacrifice after 3 weeks from injection of A2780-luc cells (control group, *n* = 8; AF, *n* = 5; Au(PEt_3_)I, *n* = 5). * *p* < 0.05, ** *p* < 0.01. Adapted from ref. [61]. Right panel Representative pseudocolor BLI images tracking A2780-luc cell emission in mice at increasing time intervals after A2780-luc injection. Color from blue (lower) to red (higher). The light intensity levels are reported as counts per minute (cpm). Adapted from ref. [61].

When both A2780 xenografted or orthotopic mouse models were treated with a dose of 15 mg/kg (three times per week for 2 weeks), the potent anticancer activity of Au(PEt_3_)I was unambiguously confirmed. This anticancer potency was even higher than that exerted by auranofin. Indeed, Au(PEt_3_)I was extremely effective after a small number of doses (less than 1 week of treatment) and capable of inducing almost complete cancer remission (see Figure 5) [61]. These features strongly warrant further preclinical and clinical studies of this promising auranofin analog.

### 2.3. Testing Novel Drug Combinations

In clinical practice, anticancer chemotherapy protocols mainly rely on the concomitant administration of different drugs, including biological or immunotherapy drugs. The simultaneous use of metal-based complexes, such as cisplatin, carboplatin, and oxaliplatin, with other drugs allows a better pharmacological outcome and could minimize the risk of resistance phenomena and recurrence [62]. Accordingly, this strategy is largely pursued in tumors that present a high rate of recurrence or that become refractory to single-agent treatments, such as ovarian (OC) or colorectal cancer (CRC). Combinations can involve molecules capable of DNA damage, drugs that target specific signaling pathways, and alkylating agents that simultaneously target multiple cancer pathways or hallmarks, eventually producing additive or synergistic anticancer activities [63]; for instance, clinical protocols for ovarian cancer widely exploit the combination of the platinum-based agents’ cisplatin or carboplatin with paclitaxel or topotecan [64,65]. In addition to the reference clinical combinations that are currently used in OC, several others have entered clinical trials, and some remain under evaluation. Table 1 summarizes chemotherapy trials in platinum-sensitive recurrent ovarian cancer.

In these novel combinations, systems and cell pathways, different from nuclear DNA, were targeted. The concomitant administration of platinum complexes with Gem and Bev, which is a monoclonal antibody able to block angiogenesis by binding to vascular endothelial growth factor (VEGF) [67], showed significant improvement in progression-free survival (PFS). Another targeted approach is the inhibition of poly (adenosine diphosphate [ADP]–ribose) polymerase (PARP). When DNA damage occurs in cells, there is the risk of large-scale loss of genetic information. PARP enzymes play a key role in restoring the integrity of the genome. Accordingly, they represent an interesting target for anticancer drugs that specifically interact with these enzymes triggering the cell death cascade. Among various drugs, olaparib, niraparib, and rucaparib have found approval in OC treatment [66,68].

Similarly, CRC is one of the most common cancers globally, and among the most diagnosed cancers in the United State and Europe for both men and women [69]. Due to these high rates of incidence, it is among the most life-threatening cancers, with mortality strongly dependent on many factors. Early diagnosis is thus extremely important, and helps to increase the effectiveness of treatments and increase the cure rate [70]. In spite of the important improvements in diagnosis, surgery, and chemotherapy protocols, thousands of patients continue to receive an adverse prognosis, with a consequent poor five-year survival rate. This applies predominantly to those patients with advanced metastatic disease. In addition to the primary tumor, liver metastasis represents a common and serious complication; thus, for decades, CRC patients with hepatic metastasis have been considered incurable. Despite recent significant improvements in available therapeutic approaches, in the presence of metastatic forms of CRC, an integrated strategy is often necessary, combining surgical resection of the tumor with chemotherapy treatments [71]. Perioperative or adjuvant chemotherapy mainly relies on platinum-based drugs such as cisplatin and its analog oxaliplatin. These drugs are administered intravenously in combination with other antiblastic drugs, such as 5-fluorouracil, leucovorin, or capecitabine, according to the different clinically established protocols [62,72]. However, both cisplatin and oxaliplatin have important problems frequently associated with treatment failure. The major disadvantages are the high systemic toxicity and the insurgence of drug resistance; the latter is an issue, in particular, in cases requiring prolonged treatments. Drug “cocktails” are being extensively investigated in an attempt to improve the clinical outcomes in CRC. Accordingly, this strategy offers an important opportunity for the discovery of novel and ameliorated drug combinations based on inorganic molecules. For instance, anti-Epidermal Growth Factor Receptor (EGFR) antibodies have been developed and are now being tested in combination with other drugs, including inorganic drugs. Pairwise combinations of inhibitors of mitogen-activated protein kinase enzymes MEK1 and/or MEK2 have been explored. Overall, attention is now being also directed to combined target therapy, allowing the selective inhibition of critical pathways [73].

**Scheme 1:**
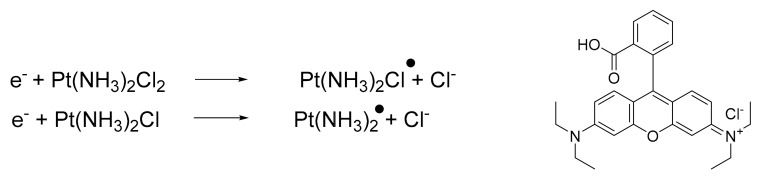
Radicals’ production from the reaction of cisplatin with an electron donor (**left**) and chemical structure of Rhodamine-B (BV10) (**right**) [74].

Additionally, it is possible to combine platinum drugs with specific molecules capable of modulating the reactivity, thus improving the final pharmacological outcome. In a recent paper, Zhang and Lu reported a novel combination of cisplatin with [9-(2-carboxyphenyl)-6-diethylamino3-xanthenylidene]-diethylammonium chloride (Rhodamine-B, BV10) [74,75]. This combination is of interest because BV10 is a molecule that is able to react with cisplatin. The products of this reaction are cisplatin radicals (Scheme 1), which are highly effective in DNA strand breaks, thus enhancing anticancer effects.

From in vitro and in vivo experiments, it was highlighted that the combination of a selected electron donor with cisplatin is exploitable for overcoming drug resistance, thus eventually widening the clinical application of cisplatin. Of note, this augmented anticancer potency was operative in different cancer cell lines, including those of ovarian and lung cancer. In addition, this combined treatment allowed the effective doses of cisplatin to be reduced, while also not resulting in additional toxic effects [74].

#### The Case of Cisplatin and Riluzole Combination Therapy

**Figure 6:**
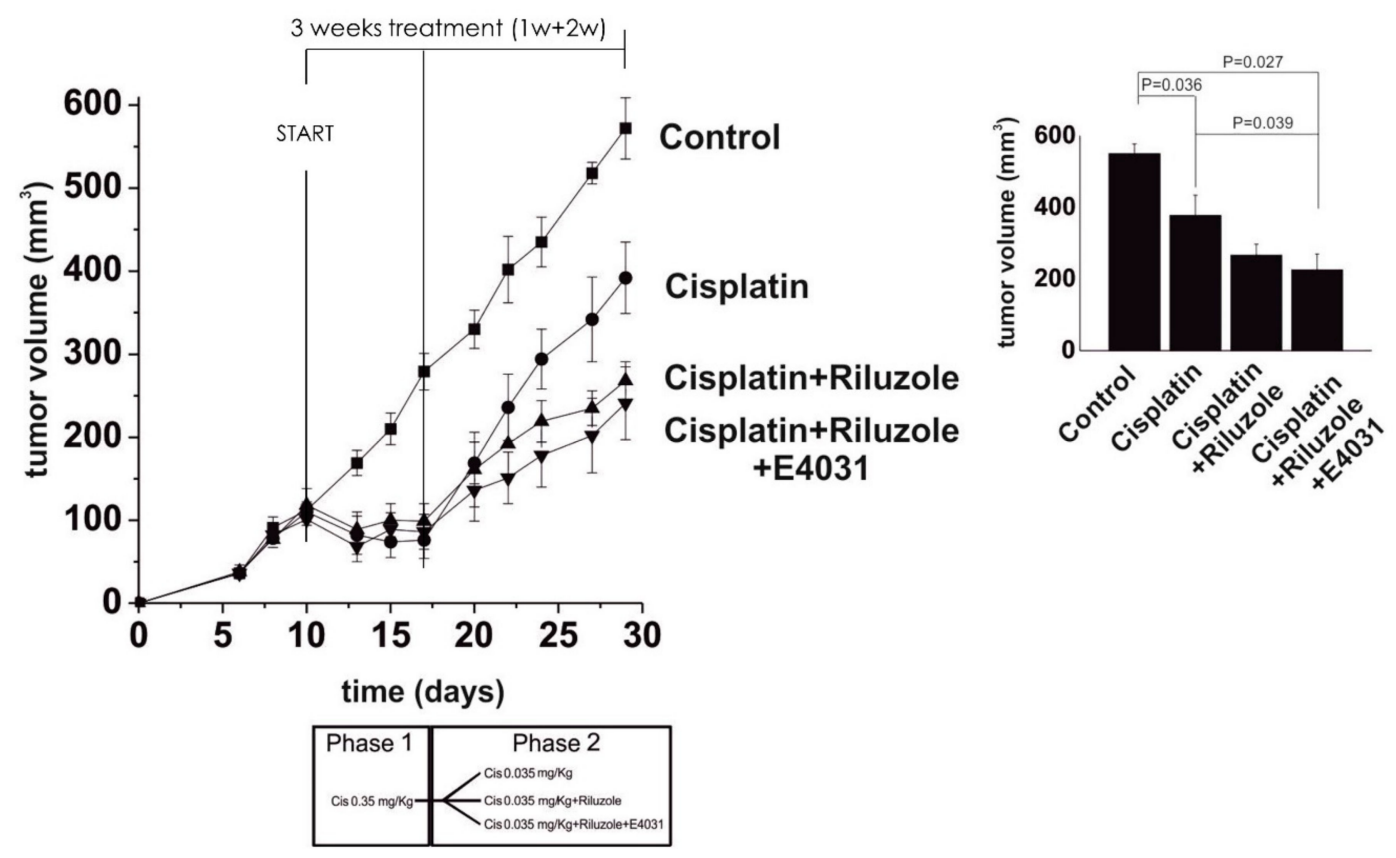
Left panel: Time course of tumor growth in control, cisplatin-, cisplatin + riluzole-, and cisplatin + riluzole + E4031-treated mice. Cisplatin was administered at 0.35 mg kg^−1^ for the first week and then lowered to 0.35 mg kg^−1^ for the following 2 weeks to mimic resistance (see the scheme of treatment at the bottom). The slopes of the curves were: cisplatin = 0.056; cisplatin + riluzole = 0.036; cisplatin + riluzole + E4031 = 0.033. Right panel: The volume of tumor masses was measured at killing and calculated by applying the ellipsoid equation. Data are reported as the mean ± s.e.m. of the number of masses shown in the figure. The onset of chemoresistance was reproduced by treating the xenografted animals with full cisplatin doses first and then with very low doses. Statistical analysis was performed by one-way ANOVA (adapted from reference [79], Attribution-Non-Commercial-(CC BY-NC-SA 4.0)).

Recently, we investigated the role that potassium channels play in the transport, uptake, and, eventually, the pharmacological effects of cisplatin and its approved analog oxaliplatin in both in vitro and in vivo CRC models. K^+^ channels are often dysregulated in cancer [76,77]. Specifically, the Ca^2+^-activated K_Ca_3.1 and the voltage-dependent K_v_11.1 channels are upregulated when the cancer is in the progression phase [78], thus contributing to its spread, but also to chemoresistance [79]. Because the sensitivity to cisplatin has been demonstrated to be dependent on K^+^ channels in several tumors [80,81], a deep investigation of the role of K^+^ channels was carried out in cisplatin-resistant CRC models to assess if cisplatin resistance could be overcome using specific K^+^ channel-modulating agents. Results revealed that compounds that activate K_Ca_3.1 (SKA-31), inhibit K_v_11.1 (E4031), or have both effects (riluzole), promote cisplatin uptake and enhance apoptosis in cisplatin-resistant cells, both in vitro and in a mice model (HCT-116 cells xenografted subcutaneously into immunodeficient nude mice). These results support the translational potential of the use of K^+^ channel modulators to overcome cisplatin resistance in CRC [79]. Remarkably, in cisplatin-resistant CRC cells, K_Ca_3.1 activators, K_v_11.1 inhibitors, and riluzole—i.e., an approved drug currently used in the treatment of amyotrophic lateral sclerosis, and that entered clinical trials as an anticancer drug [79]—showed synergistic action with cisplatin. Indeed, they were capable of re-establishing the pro-apoptotic and cytotoxic effects of cisplatin, even at very low doses (Figure 6).

It is important to stress that the effects of the tested K^+^ channel-modulators on apoptosis and the cell cycle were higher in cisplatin-resistant cell lines. Of relevance, it was found that the same effects were also evident for oxaliplatin, thus confirming the translational potential of the data. Overall, these results confirm that the innovative combination of cisplatin, or its analogs, with riluzole could be effective to overcome the challenging problem of resistance. Additionally, such a combination appears effective in allowing the dose of cisplatin that is pharmacologically effective to be reduced, thus diminishing, at least in principle, its side effects.

### 2.4. Newly Synthesized Complexes Coupling Different Anticancer Drugs

As mentioned previously, some of the major drawbacks related to the platinum-based anticancer treatments are the well-known and severe side effects (e.g., oto- and nephro-toxicity) often associated with the insurgence of intrinsic and acquired resistance to these therapies [82,83,84]. Hence, the incidence of drug resistance, associated with tumor metastasis, represents one of the major challenges in the discovery of effective chemotherapeutic treatment. Several combinatorial therapies have been extensively studied and applied in clinical practice [85,86]. Another promising strategy explored in recent years considers the coupling of two or more drugs in a single molecule. This strategy has the additional advantage of allowing better control of the pharmacokinetics, biological fates, and co-localization properties of the drugs, and the resulting complexes may be multifunctional [87].

**Figure 7:**
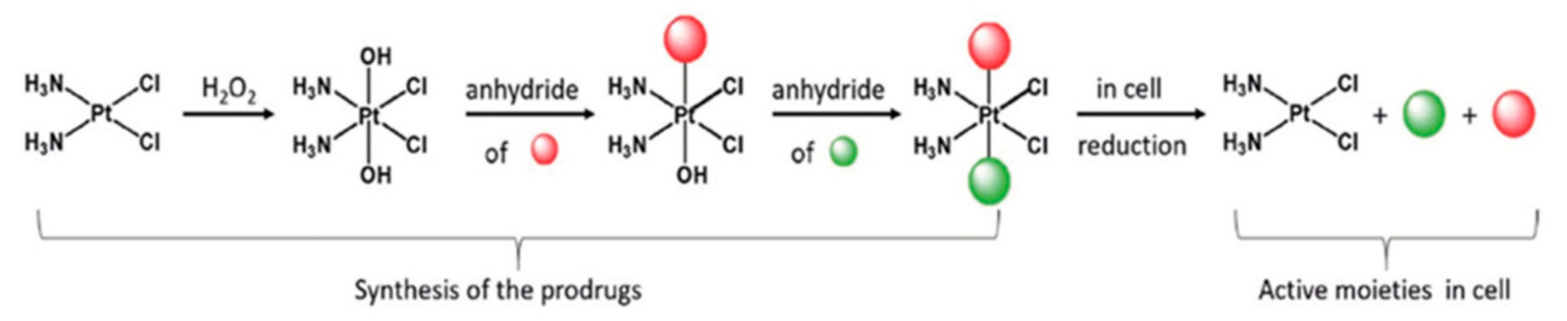
Synthetic strategy and mode of action of Pt(IV) prodrugs proposed by Gibson and coworkers. The axial position can be easily functionalized with two different biologically active molecules. Adapted from ref. [89].

**Figure 8:**
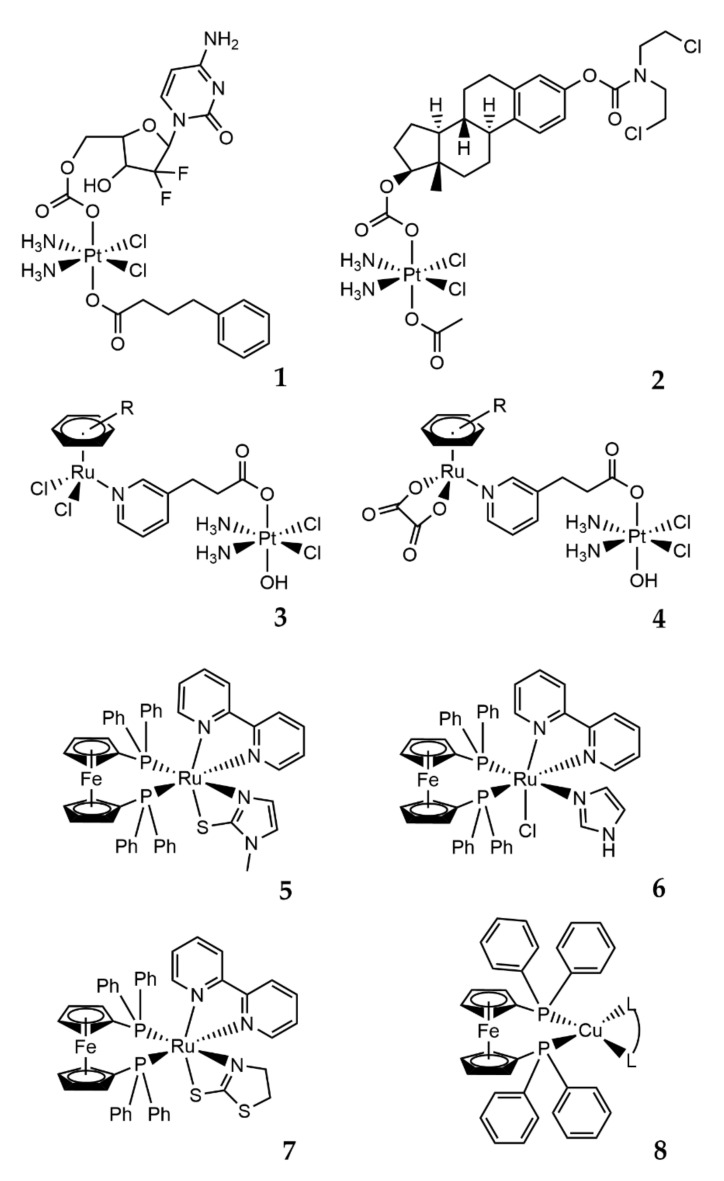
Some representative metal-based complexes are coupled with bioactive molecules (**1**,**2**) and heterobimetallic cytotoxic compounds (**3**–**8**).

The use of Pt(IV) complexes as prodrugs is now recognized as a simple and effective strategy to overcome the shortcomings of Pt(II) drugs (intravenous administration, low bioavailability, severe side effects, and acquired resistance) [88,89,90]. The concept behind the strategy is that the fundamental square-planar Pt(II) complexes (i.e., cisplatin, carboplatin, and oxaliplatin) can be chemically oxidized to their octahedral Pt(IV) counterparts [91], as depicted in Figure 7. In this way, the two axial coordinative positions of the Pt(IV) center can be functionalized with a plethora of bioactive molecules, such as gemcitabine, estramustine (Figure 8; compounds **1**, **2**), and paclitaxel [89], but also some anti-inflammatory molecules [92], estrogens [93], etc. A small number of relevant Pt(IV) complexes have been summarized in a recent review by Lippard and coworkers [7].

This strategy takes advantage of the chemical inertness of the functionalized platinum(IV) complexes towards biological targets until being reduced by chemical agents (including biomolecules) or UV-Vis exposure [94]. After the reduction, the reactive Pt(II) compound is released, in addition to the other two axially bound bioactive molecules [89]. Other interesting and promising heterobimetallic complexes based on the Pt(IV) analog of cisplatin have been developed. Zhu and coworkers reported elegant synthesis and evaluations of a series of water-soluble Pt(IV)–Ru(II) heterodinuclear complexes (Figure 8; compounds **3**, **4**), designed as ruthplatins, aiming to take advantage of both metals to obtain bifunctional anticancer drug candidates [95]. These molecules, bearing a functionalized Pt(IV) cisplatin analog and different Ru(II)-arene moieties in the apical positions, revealed elevated cytotoxicity, particularly in 2D and 3D tumor models of cisplatin-resistant cancer cells, in addition to an impressive ability to suppress the migration of tumor cells [95].

Due to the antimetastatic activity, high selectivity, and cytotoxicity for human tumor cell lines, ruthenium(II) complexes are highly attractive, and combinations with other metal centers lead to new heterobimetallic complexes with appealing anticancer properties. This is the case of some Ru(II)/Fe(II) complexes described by Batista (Figure 8; compounds **5**–**7**) [96], and the reported results show high cytotoxicity and selectivity indexes (selectivity index = IC_50_ value in the normal cell line/IC_50_ value in the cancer cell line) for the human triple-negative breast tumor cell line (MDA-MB-231). The study of the mechanism of cell death induced in the MDA-MB-231 line indicated an apoptotic pathway. Moreover, for these complexes, multiple targets of action were identified: induction of DNA damage, mitochondrial depolarization with a reduction in mitochondrial membrane potential, increase in reactive oxygen species levels, and increased expression levels of caspase 3 and p53. In addition, inhibition of angiogenesis caused by MDA-MB-231 tumor cells in the chicken chorioallantoic membrane (CAM) model was evidenced [96,97].

Recently, other metal centers and their combinations were also evaluated as potential anticancer agents, although they have not yet been studied closely for biological applications. One interesting combination is represented by new complexes bearing one Fe and one Cu center. In these molecules, the chelating 1,10-bis(diphenylphosphino)ferrocene (dppf) ligand combines the ferrocene properties with the coordination properties of tertiary phosphines towards Cu(I) (Figure 8; compound **8**) [98]. The resulting Cu(I)–dppf complexes have attracted interest for anticancer applications, showing considerable cytotoxicity towards several cancer cells [99,100]. In addition, Morais and coworkers recently reported the potential of copper(I)-phosphane compounds with several heteroaromatic ligands as therapeutic agents against ovarian (A2780) and breast (MCF7) adenocarcinomas [101]. The described molecules are the first studies reported in the literature related to the evaluation of Cu(I)–dppf complexes as cytotoxic anticancer agents. The compounds showed cytotoxic potential in both MCF7 and MDAMB231 human breast cancer cell lines following 24 h of treatment. Notably, these Fe/Cu compounds were found to be up to 76 times more cytotoxic than cisplatin in the MCF7 cells [98].

Numerous other examples describing the development and applications of several new molecules combining Pt, Ru, Au, and Ti in heterobimetallic complexes with promising biological properties can be found in the literature [87,102,103,104,105,106].

#### The Case of Arsenoplatin-1

In addition to the successful use of platins for the treatment of several solid tumors, other small inorganic molecules have gained increasing attention from the scientific community as effective drugs against blood tumors, such as leukemia. This is the case of arsenic trioxide (Trisenox), which has been successfully applied in clinical practice for the treatment of acute promyelocytic leukemia [107,108].

However, patients who receive this molecule also suffer from several side effects, particularly those individuals with comorbidities, such as older adults and patients with cardiac dysfunction or other organ dysfunctions. The adverse effects reported principally concern the high blood arsenic levels that can lead to arrhythmia [109].

Although the mechanism of action of arsenic trioxide is still poorly understood, it is accepted that its efficacy is related to the presence of As(III) species. However, once administered, As(III) can undergo interconversion to As(V), making the treatment less effective [110]. Furthermore, accurate control of arsenic species is recommended because of its intrinsic high toxicity. This is among the limiting aspects of the use of arsenic in leukemia chemotherapy. Moreover, limited efficacy of As_2_O_3_ has been observed in the treatment of solid tumors [111] due to rapid renal clearance, thus impairing its uptake in tumors [112]. Several attempts have been made to overcome these toxic effects and enhance the bioavailability by exploiting new strategies, such as biocompatible human serum albumin coated arsenic trioxide nanoparticles [112,113], encapsulation with nanoliposomes [109], and a metal-organic framework (MOF) as a drug carrier material [114].

**Figure 9:**
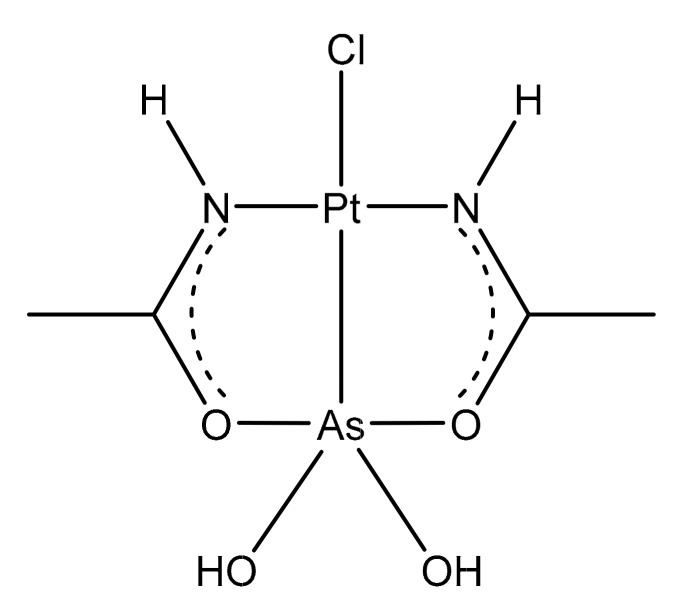
Chemical structure of arsenoplatin-1 (AP-1).

Recently, we described the biological properties of a new molecule, arsenoplatin-1 (AP-1, Figure 9), containing an arsenous acid moiety covalently linked to a platinum(II) center and characterized by an unusual five-coordinate As(III) geometry [115].

AP-1 was designed and synthesized with the aim of coupling the properties of cisplatin and trisenox in a single molecule to improve the pharmacological action. This dual pharmacophore agent was able to trigger apoptotic cell death through the platinum, which is able to bind the nuclear DNA [116], and, at the same time, via the As(III)-containing portion, which is capable of the thiophilic binding of cysteine-rich proteins. Furthermore, the As center can also act on zinc-finger-containing regulatory proteins due to its ability to displace zinc [117].

**Figure 10:**
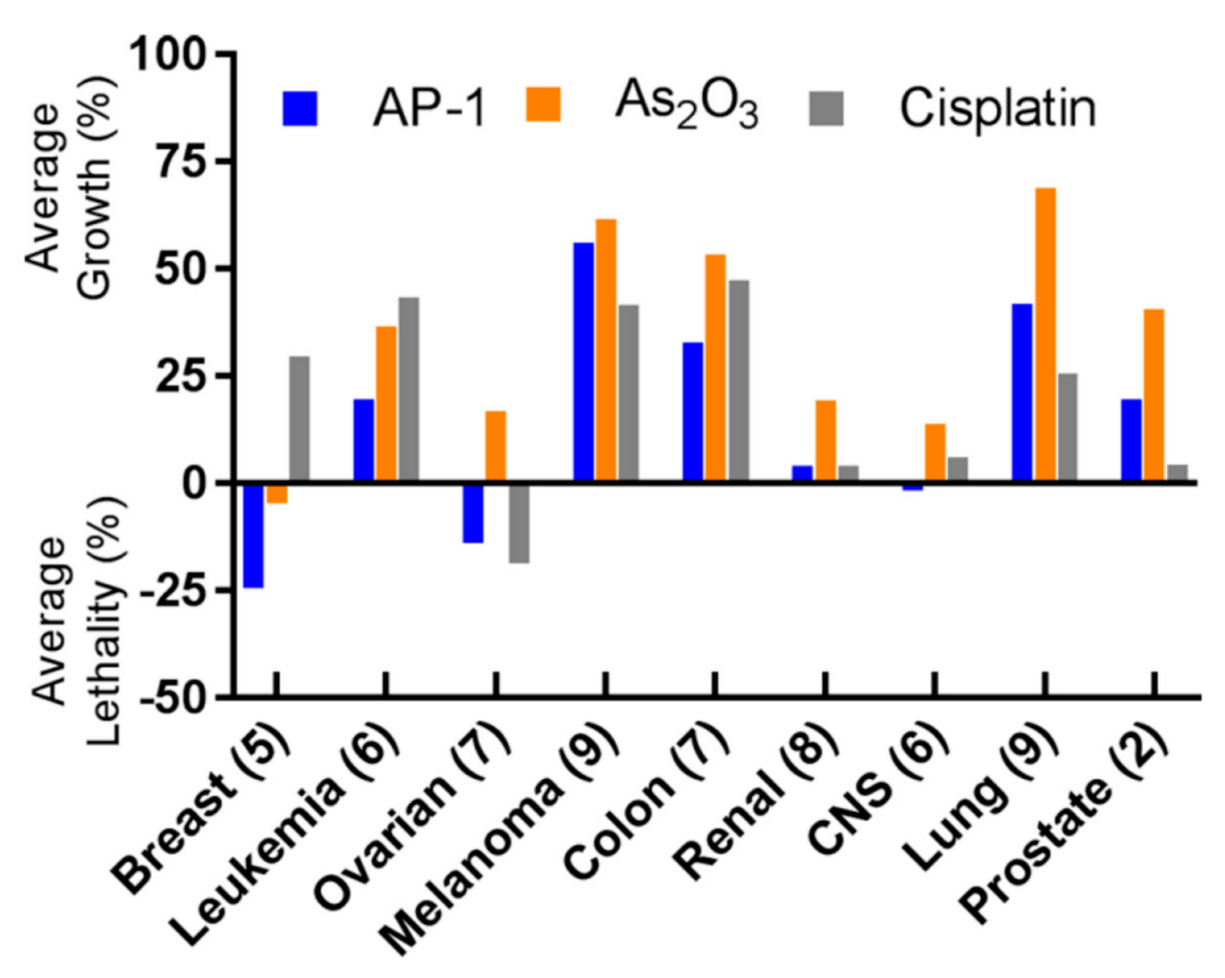
Summary of the NCI-60 human tumor cell line screen. AP-1 shows superior activity compared to arsenic trioxide in all nine indications tested; it also shows higher potency than cisplatin in breast, leukemia, colon, and CNS. Numbers in parentheses represent the number of cell lines tested for each indication, and the one-dose data are reported as a mean graph of the percentage growth of treated cells, according to the NCI-60 form. The cell growth in the one-dose assay is relative to the no-drug control and relative to the time zero number of cells, allowing the detection of both cellular growth inhibition (values between 0 and 100) and cellular lethality (values less than 0). (Reprinted with permission from reference [115]. Copyright 2019, American Chemical Society).

Preliminary in vitro cytotoxicity evaluations suggested that arsenoplatins act with a distinct mode of action in comparison to cisplatin and As_2_O_3_, showing an ability to overcome platinum resistance mechanisms [118]. AP-1 was challenged with the NCI-60 panel of human tumor cell lines, showing a remarkable anticancer profile. Again, when compared to the cisplatin activity, AP-1 showed greater cytotoxic potential towards the breast, leukemia, colon, and CNS cancer cell lines, and was comparable to cisplatin in ovarian and renal cancer cell lines [115]. The comparison between the activity of AP-1 and those of cisplatin and As_2_O_3_ in the NCI-60 panel is shown in Figure 10.

Other tests on the anticancer activity of AP-1 were directed to highlight its mechanism of action and to the understanding of an eventual underlying cooperative effect between platinum and arsenic pharmacophores. The authors firstly used the method reported by Chou [119] to test for synergy between single AP-1 components, i.e., arsenic trioxide and cisplatin, in a highly aggressive triple-negative breast MDA-MB-231 cancer cell line resistant to cisplatin. The results indicate a significant synergistic action between the two drugs at all the tested ratios; however, with cisplatin to arsenic trioxide ratio of 1:1, the optimal effects were achieved. Subsequently, AP-1 was challenged against the MDA-MB-231 cancer cell line. The data obtained after 8 h of exposure showed a higher cytotoxic potential (IC_50_ = 9.5 ± 0.3 μM) compared to cisplatin (IC_50_ = 22 ± 0.5 μM).

The Pt:DNA ratio for cell lines treated with AP-1 was 1.1, and a very similar As:Pt ratio (0.97:1) was also revealed, suggesting that the As-Pt bond in AP-1/DNA adducts remain unaltered, at least in the early stage of adduct formation. Subsequently, the As(OH)_2_ moiety was released from the AP-1/DNA adduct, contributing to the overall toxicity of AP-1 across different mechanisms, such as the binding with sulfur-containing proteins [120]. Using high-resolution mass spectrometry, O’Halloran and coworkers successfully demonstrated that AP-1 was able to produce stable adducts towards two selected model proteins, i.e., HEWL and bovine pancreatic ribonuclease A (RNase A) [115]. ESI-MS measurements offered clear evidence of the formation of adducts in which one or more [AP-1]^+^ fragments are associated with these proteins. The nature of these adducts was confirmed by a deep crystallographic analysis that revealed that adduct formation involves a loss of chloride followed by platinum ligation to surface-exposed histidine residues, leaving the Pt−As bond intact in the protein adducts. By comparison, the previously discussed studies of AP-1/DNA adducts in the MDA-MB-231 cancer cell line were consistent with a gradual release of the As(OH)_2_ moiety. Hence, from this experimental evidence, we can argue that arsenoplatin-1 acts as a unique and dual pharmacophore anticancer agent, showing the potential to simultaneously deliver platinum and arsenic species to a variety of hematological and solid cancers.

Recently, encouraging attempts have been made to hamper the selectivity of AP-1 for cancer cells. One promising strategy involves horse spleen apo-ferritin nanocages, which can be used to encapsulate arsenoplatins, leading to an effective improvement of the selectivity for the A431 (human epidermoid carcinoma) and SVT2 (murine BALB/c-3T3 fibroblasts transformed with SV40 virus) cancer cells with respect to HaCaT (immortalized human keratinocytes) and Balb/c-3T3 (immortalized murine BALB/c-3T3 fibroblasts) healthy cells [121].

## 3. Conclusions

In this review, we discuss strategies for the development of novel inorganic molecules with medicinal applications. Metal-based drugs could be a valuable source of active molecules in the field of medicinal chemistry. Due to the presence of the metal center, their properties cannot be easily reproduced by organic molecules, making metal-based compounds an alternative family of active compounds with respect to organic drugs. Accordingly, it is extremely important to optimize their design and, in general, the development process. This goal can be achieved by selecting the most reliable and valuable approaches that combine reasonable cost and effectiveness, while also being less time-consuming. The outcomes of this research could be improved with a positive impact even from the translational perspective. The four methodologies considered here potentially serve to improve the success rate of the discovery process of metal-based drugs. In addition, by integrating the increasing mechanistic information available from modern analytical, speciation, and biological techniques with the proper strategy, it is possible to finely tune the chemical and biological features of the novel metallodrugs to develop effective and convenient personalized medicine. Finally, it is important to highlight that all of the above considerations might enhance the interest of pharmaceutical companies in inorganic molecules.

## Figures and Tables

**Table 1:** Landmark chemotherapy trials in platinum-sensitive recurrent ovarian cancer involving platinum-based anticancer drugs with outcome details (adapted with permission from ref. [66]).

Name of the Study	Primary Outcome	Study Groups	PFS (Months)	HR (95% CI)	*p*-Value	OS (Months)	HR (95% CI)	*p*-Value
ICON4	OS	Carbo	10	0.76 (0.66–0.89)	0.0004	24	0.82 (0.69–0.97)	0.02
		Carbo/Taxol	13			29		
AGO	PFS	Carbo	5.8	0.72 (0.58–0.90)	0.003	17.3	0.96 (0.75–1.23)	0.73
		Carbo/Gem	8.6			18.0		
CALYPSO	PFS	Carbo/Taxol	9.4	0.82 (0.72–0.94)	0.005	30.7	0.99 (0.85–1.16)	0.94
		Carbo/PLD	11.3			33.0		
OCEANS	PFS	Carbo/Gem/Placebo	8.4	0.48 (0.39–0.61)	<0.0001	32.9	0.95 (0.77–1.17)	0.65
		Carbo/Gem/Bev	12.4			33.6		

Abbreviations: Carbo = carboplatin; PFS = progression-free survival; HR = hazard ratio; OS = overall survival; Gem = gemcitabine; PLD = pegylated liposomal doxorubicin; Bev = bevacizumab.

## Data Availability

Not applicable.
